# Acropetal Auxin Transport Inhibition Is Involved in Indeterminate But Not Determinate Nodule Formation

**DOI:** 10.3389/fpls.2018.00169

**Published:** 2018-02-15

**Authors:** Jason L. P. Ng, Ulrike Mathesius

**Affiliations:** Division of Plant Sciences, Research School of Biology, Australian National University, Canberra, ACT, Australia

**Keywords:** auxin, indeterminate, determinate, auxin transport inhibitor, acropetal, legume, nodule

## Abstract

Legumes enter into a symbiotic relationship with nitrogen-fixing rhizobia, leading to nodule development. Two main types of nodules have been widely studied, indeterminate and determinate, which differ in the location of the first cell division in the root cortex, and persistency of the nodule meristem. Here, we compared the control of auxin transport, content, and response during the early stages of indeterminate and determinate nodule development in the model legumes *Medicago truncatula* and *Lotus japonicus*, respectively, to investigate whether differences in auxin transport control could explain the differences in the location of cortical cell divisions. While auxin responses were activated in dividing cortical cells during nodulation of both nodule types, auxin (indole-3-acetic acid) content at the nodule initiation site was transiently increased in *M. truncatula*, but transiently reduced in *L. japonicus*. Root acropetal auxin transport was reduced in *M. truncatula* at the very start of nodule initiation, in contrast to a prolonged increase in acropetal auxin transport in *L. japonicus*. The auxin transport inhibitors 2,3,5-triiodobenzoic acid and 1-*N*-naphthylphthalamic acid (NPA) only induced pseudonodules in legume species forming indeterminate nodules, but failed to elicit such structures in a range of species forming determinate nodules. The development of these pseudonodules in *M. truncatula* exhibited increased auxin responses in a small primordium formed from the pericycle, endodermis, and inner cortex, similar to rhizobia-induced nodule primordia. In contrast, a diffuse cortical auxin response and no associated cortical cell divisions were found in *L. japonicus*. Collectively, we hypothesize that a step of acropetal auxin transport inhibition is unique to the process of indeterminate nodule development, leading to auxin responses in pericycle, endodermis, and inner cortex cells, while increased auxin responses in outer cortex cells likely require a different mechanism during the formation of determinate nodules.

## Introduction

Many legume species form a symbiosis with nitrogen-fixing rhizobia, resulting in the development of nodules in the host roots. There are variations in the organogenesis of nodules in different species of legumes. Two main nodule types have been reported in the literature, indeterminate and determinate nodules. The model legume *Medicago truncatula* forms indeterminate nodules, while another model legume, *Lotus japonicus*, forms determinate nodules. Whereas indeterminate nodules involve re-initiation of cell divisions in the pericycle, endodermis, and inner cortex of the root, determinate nodules are characterized by cell divisions mainly in the middle and outer cortex, with some contributions from the pericycle and endodermis (Hirsch, 1992). Another difference between the two nodule types is that indeterminate nodules are typically elongate and maintain an apical nodule meristem; determinate nodules lose the meristematic activity of the nodule meristem and are typically round (Hirsch, 1992). So far it is not known what determines the difference between these two organogenesis programs.

Previous studies have shown that the plant hormone auxin is essential for the initiation of cell divisions in plants and that auxin gradients accompany the formation of new plant organs (Benková et al., 2003). This also seems to be the case for nodule initiation, where increased auxin responses have been localized to nodule primordia in various legume species forming both indeterminate or determinate nodules (van Noorden et al., 2007; Takanashi et al., 2011; Suzaki et al., 2012; Turner et al., 2013).

Auxin represents a group of structurally related phytohormones. The most frequently studied auxin is indole-3-acetic acid (IAA). Auxins can be divided into active and inactive/storage forms, with the latter being the more abundant form in plants (Korasick et al., 2013). Active auxins, such as IAA, activate auxin-response genes, whose gene products regulate cell division and organ growth. Other active auxins include indole-3-butyric acid (IBA), 4-chloro-indole-3-acetic acid (4-Cl-IAA), and phenylacetic acid (PAA). Each of these active auxins can be inactivated by forming conjugates with amino acids (Korasick et al., 2013). For example, IAA-Alanine and IAA-Leucine have been suggested to be IAA storage forms in *Arabidopsis thaliana* (Kowalczyk and Sandberg, 2001; Novák et al., 2012), while IAA-Asp might lead to auxin degradation and inactivation (Korasick et al., 2013). IAA-Tryptophan, interestingly, was postulated to be an auxin antagonist, inhibiting IAA action (Staswick, 2009). The interconversion between free and conjugated IAA provides one mechanism to fine tune plant development through the spatio-temporal control of active IAA concentrations.

Auxin is transported within plants via two mechanisms – a passive, long distance auxin transport system through the phloem and an active, local cell-to-cell auxin transport machinery. Active auxin transport control plays an important role during root development. It is controlled by a suite of auxin transport carriers and cellular regulators that control their intracellular abundance and localization (Overvoorde et al., 2010). In particular, members of the auxin exporter family *PIN* (*PINFORMED*) and auxin importer family *AUX1*/*LAX* (*AUXIN RESISTANT1*/*LIKE AUX1*) have been shown to contribute to the formation of auxin gradients in *Arabidopsis* (Overvoorde et al., 2010). In *M. truncatula*, expression of the *PIN* auxin efflux carriers is altered during symbiotic interactions with *Sinorhizobium meliloti*. The expression of *PIN2*, *4*, and *10* was upregulated within 24 h in response to *S. meliloti* inoculation or Nod factor treatment (Plet et al., 2011; Ng et al., 2015). Knockdown of *PIN2*, *3*, and *4* reduced nodule numbers on transgenic *M. truncatula* roots (Huo et al., 2006). *In situ* hybridization of *M. truncatula LAX1* (homolog of the *Arabidopsis* auxin influx carrier *AUX1*) mRNAs suggested that these auxin influx carriers are involved in early nodule primordia development and vasculature differentiation (de Billy et al., 2001). Such evidence strongly supports the role of auxin transport during nodule development.

Computer modeling suggested that during the early stages of *M. truncatula*–*S. meliloti* symbiosis, a temporary decrease in acropetal auxin efflux is the most plausible mechanism to explain the observed patterns of auxin accumulation in the dividing cells that comprise a nodule primordium, although auxin import is also likely to contribute (Deinum et al., 2012; Roy et al., 2017). When changes in acropetal auxin transport through the stele were combined with a hypothesized diffusible signal from the epidermis – mimicking a signal from infecting rhizobia – auxin accumulation occurred in cells where auxin responses have been localized in *M. truncatula*, i.e., pericycle, endodermis, and inner cortex (Deinum et al., 2016). Interestingly, a temporary decrease in auxin efflux has so far only been documented for indeterminate nodules, such as those forming on *M. truncatula*, white clover, and vetch (Mathesius et al., 1998; Boot et al., 1999; van Noorden et al., 2006). This auxin transport inhibition was absent in the nodulation defective *cre1 (cytokinin response 1)* mutant of *M. truncatula* that has a mutation in a cytokinin receptor (Ng et al., 2015). In this mutant, auxin transport inhibitors (ATIs) could restore both auxin transport inhibition as well as nodule initiation and auxin responses, suggesting that auxin transport inhibition in *M. truncatula* is required for correct auxin localization in the pericycle, endodermis, and inner cortex, which then leads to their divisions (Ng et al., 2015). Pacios-Bras et al. (2003) reported a temporary increase in auxin transport in response to Nod factor treatment in *L. japonicus*, although this was not statistically analyzed. Based on findings that lack of flavonoids in *M. truncatula* abolished auxin transport control and nodulation by rhizobia (Wasson et al., 2006), but lack of auxin-transport reducing isoflavonoids in soybean could be compensated for by addition of nod-gene inducers to infecting Bradyrhizobia (Subramanian et al., 2006), it was suggested that auxin transport control may be specific to indeterminate nodulation (Subramanian et al., 2007). However, no detailed auxin transport measurements have been published in other determinate nodule-forming species. It therefore remains unclear whether auxin transport inhibition is likely a mechanism leading to auxin accumulation in inner cortical cells in indeterminate nodule-forming species, but not in determinate nodule-forming species where auxin accumulation is localized in the middle/outer cortex.

Experiments using synthetic ATIs, such as *N*-1-naphthylphthalamic acid (NPA) and 2,3,5-triiodobenzoic acid (TIBA), have been used to uncover the roles of polar auxin transport in plant development, including lateral root organogenesis, inflorescence growth, and meristem maintenance (Casimiro et al., 2001; Scanlon, 2003; Wu and McSteen, 2007). Intriguingly, the application of ATIs on the roots of indeterminate nodule-forming legumes, such as *M. truncatula*, alfalfa, pea, and white sweet clover, can induce the formation of nodule-like structures, broadly termed pseudonodules (Hirsch et al., 1989; Scheres et al., 1992; Wu et al., 1996; Rightmyer and Long, 2011). Pseudonodules have a globular external structure resembling nodules, but contain undifferentiated cortical, endodermal, and pericycle cells. In addition, they fail to develop the characteristic peripheral vasculature (Guan et al., 2013) and do not house rhizobia. The detailed structure of early developmental stages of pseudonodules is not well described in the literature. On the contrary, ATIs failed to form pseudonodules on the roots of *L. japonicus* (Kawaguchi et al., 1996; Takanashi et al., 2011) and have only been reported to induce nodule-like structures in one species forming determinate nodules, *Macroptilium atropurpureum* (siratro), albeit with no description of the structure (Relić et al., 1993). This suggests that auxin transport inhibition is a mechanism inducing nodule structures by localizing auxin in the inner cortical region in indeterminate- but likely not determinate-nodule forming species (Kohlen et al., 2017).

Here, we aimed to compare changes in auxin transport and localization during indeterminate and determinate nodule formation. We compared acropetal (toward the root tip) auxin transport in corresponding root segments of *M. truncatula* and *L. japonicus* seedlings. We corroborated these findings with localization of auxin responses during nodulation and with direct quantification of auxin concentrations in these root segments, as we hypothesized that auxin transport affects the available pool of auxin (active and conjugated) at the nodule initiation site. We also tested the ability of ATIs to induce pseudonodules in a number of legume species forming indeterminate and determinate nodules, as well as their ability to inhibit auxin transport and induce auxin responses in the root.

## Results

### Auxin Transport Regulation during Nodulation Differs between *Medicago truncatula* and *Lotus japonicus*

**FIGURE 1:**
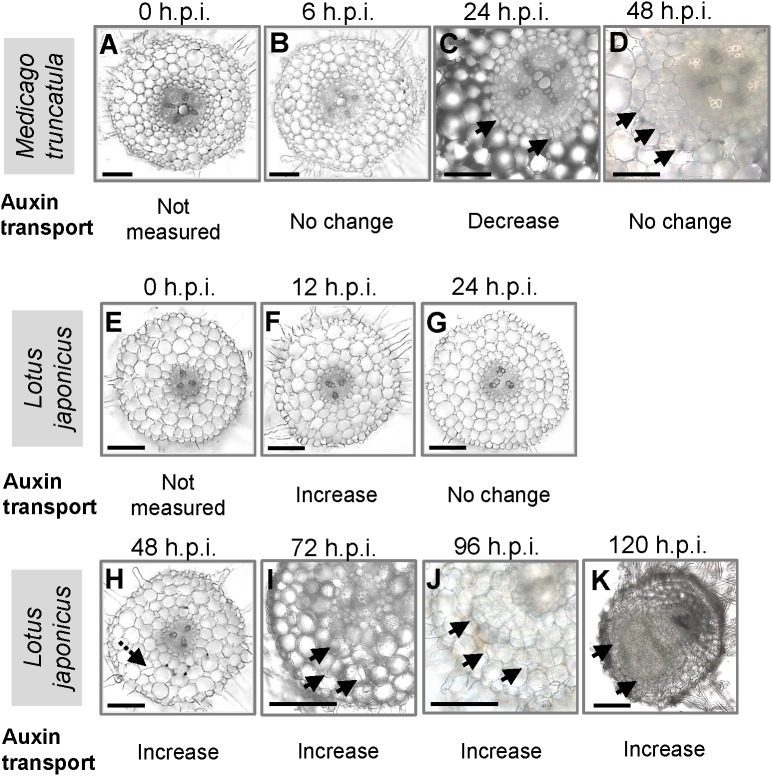
Nodule development in *Medicago truncatula* and *Lotus japonicus*. Cross sections of roots showing the stages of nodulation at **(A)** 0, **(B)** 6, **(C)** 24, and **(D)** 48 h post-inoculation (h.p.i.) in *M. truncatula*; cross sections of roots showing the stages of nodulation at **(E)** 0, **(F)** 12, **(G)** 24, **(H)** 48, **(I)** 72, **(J)** 96, and **(K)** 120 h.p.i. in *L. japonicus*. Changes in acropetal auxin transport at each individual stage are summarized below each figure. At least 10 roots were examined at each stage. Black arrows indicate pericycle, endodermal, and cortical cell divisions. Dotted black arrow in **H** indicates a possible cortical cell division. Scale bars represent 200 μm.

We were interested to compare auxin transport at the equivalent nodule developmental stages in *M. truncatula* and *L. japonicus* during the early stages of nodulation. To coordinate the time point of measurement with the onset of nodule development, we made root cross sections at different nodule developmental stages (**Figure 1**). In *M. truncatula*, no visible cell divisions were detected at 0 and 6 hours post-inoculation (h.p.i.) (**Figures 1A,B**). We observed the first cortical cell divisions as early as 24 h.p.i. (**Figure 1C**) and more extensive cortical, endodermal, and pericycle divisions at 48 h.p.i. (**Figure 1D**). In *L. japonicus*, no cell divisions were detected at 0, 12, or 24 h.p.i. (**Figures 1E–G**). In one root, we found a single cortical cell division at 48 h.p.i., but not in any other roots. The first visible cell divisions appeared at 72 h.p.i. (**Figure 1I**) and visible nodule primordia at 120 h.p.i. in the majority of roots (**Figure 1K**).

**FIGURE 2:**
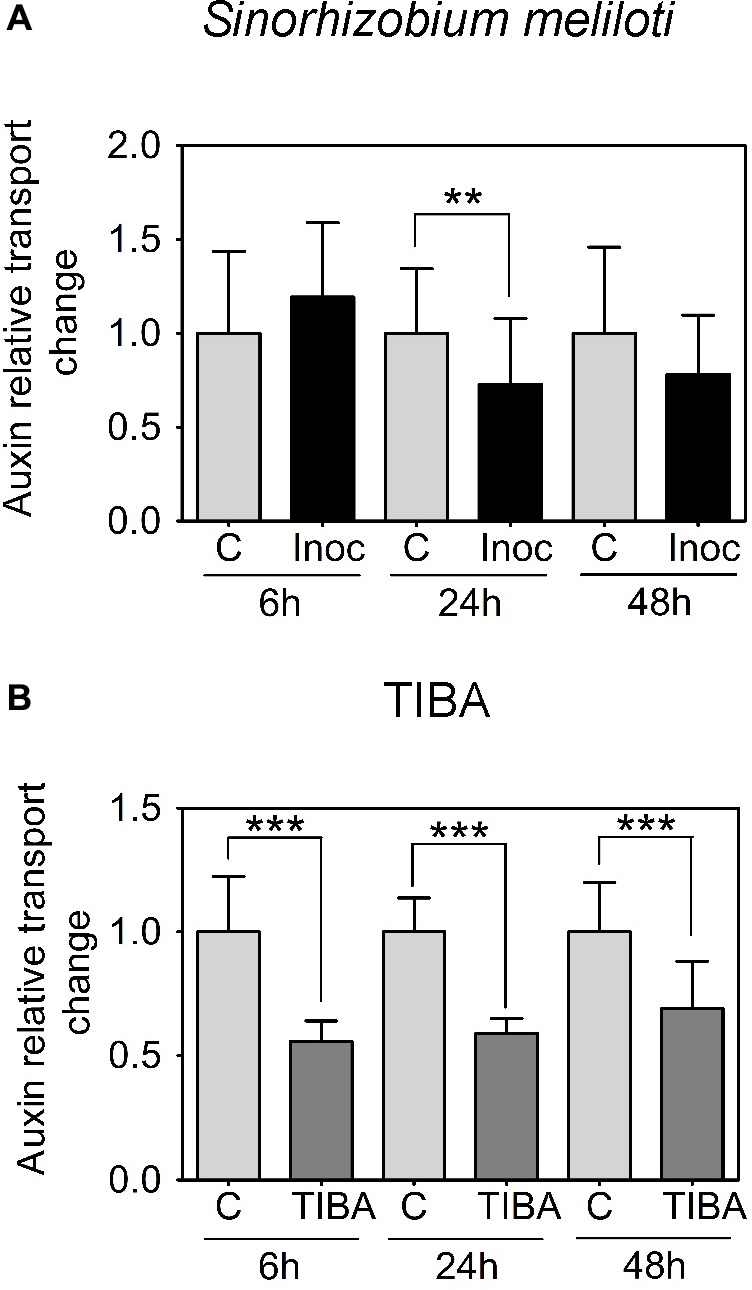
Acropetal auxin transport measurements of *M. truncatula* root segments. **(A)** Relative auxin transport changes were measured in mock or *Sinorhizobium meliloti*-spot inoculated root segments at 6, 24, and 48 h.p.i. **(B)** Relative acropetal auxin transport changes in mock- or TIBA-flood-treated root segments at 6, 24, and 48 h post-treatment. Mann–Whitney *U*-tests and unpaired *t*-tests were used for statistical analyses in **A** and **B**, respectively (*p* < 0.05, *n* = 15–25 per treatment or time point). Asterisks indicate statistically different auxin relative transport change (^∗∗^, very significant; ^∗∗∗^ extremely very significant). Graphs show mean and SD. Abbreviations: C, control; Inoc, inoculated; and TIBA, 2,3,5-triiodobenzoic acid.

We were interested in auxin transport regulation prior to the formation of visible nodule primordia on the roots. In *M. truncatula*, we measured acropetal auxin transport in the segment just below the inoculation spot at 6, 24, and 48 h.p.i. Auxin transported into the segment below the site of nodule initiation was chosen because if dividing cells inhibit the acropetal export of auxin this would be detectable as a reduced amount of auxin transported into the segment just below these cells. Consistent with our previous findings (Ng et al., 2015), we measured a significant decrease in acropetal auxin transport into the segment below the inoculation site at 24 h.p.i., but not at 6 or 48 h.p.i. (**Figure 2A**).

**FIGURE 3:**
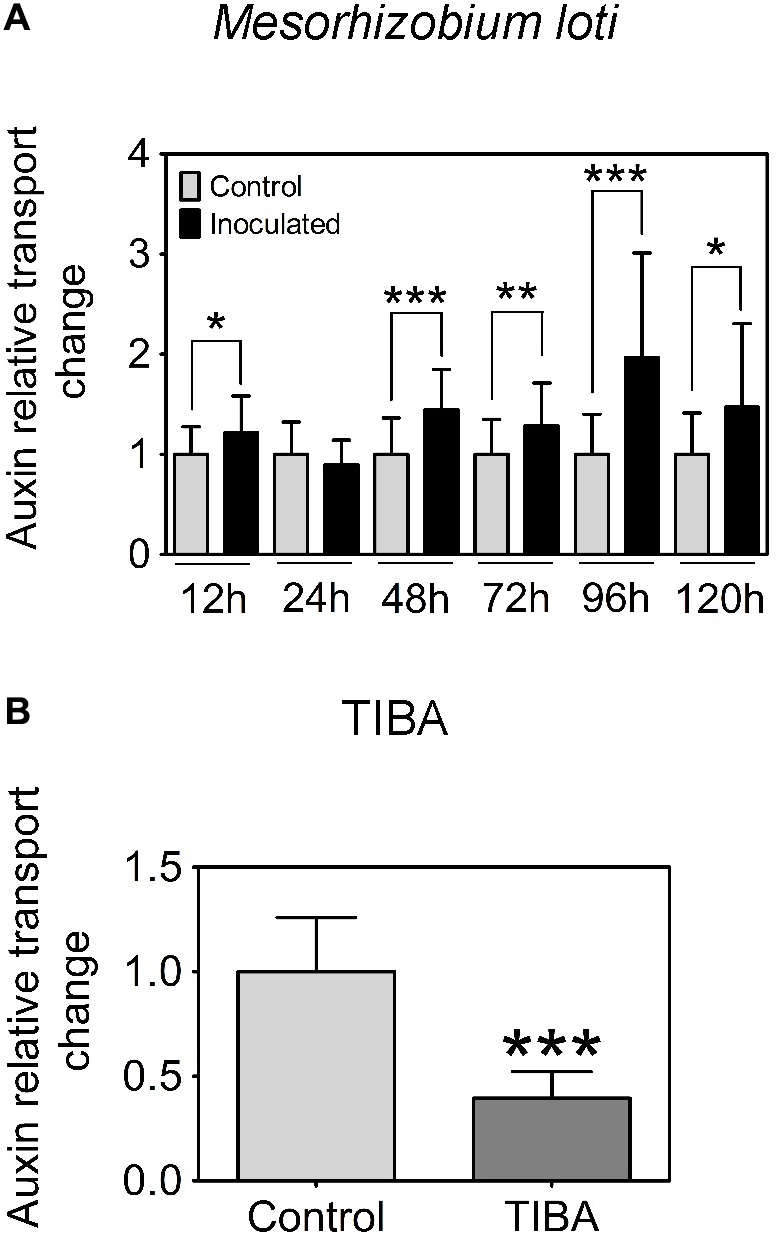
Acropetal auxin transport measurements of *L. japonicus* root segments. **(A)** Relative auxin transport changes were measured in mock or *Mesorhizobium loti*-spot inoculated root segments at 12, 24, 48, 72, 96, and 120 h.p.i. **(B)** Relative auxin transport changes were measured in mock- or TIBA-treated root segments at 48 h post-treatment. Unpaired *t*-tests were used for statistical analyses (*p* < 0.05, *n* = 25–30 per treatment or time point). Asterisks indicate significant change in relative auxin transport (^∗^, significant; ^∗∗^, very significant; ^∗∗∗^ extremely very significant). Graphs show mean and SD. Abbreviation: TIBA, 2,3,5-triiodobenzoic acid.

In *L. japonicus*, visible nodule primordia only formed after 120 h.p.i. under our growth conditions (**Figure 1K**). Hence, we measured auxin transport at 12, 24, 48, 72, 96, and 120 h.p.i. Similar to the findings reported in Pacios-Bras et al. (2003), we found a significant increase in acropetal auxin transport at 48 h.p.i. in the segment below the rhizobia inoculation spot (**Figure 3A**). In addition, acropetal auxin transport also increased significantly at 12, 72, 96, and 120 h.p.i. (**Figure 3A**).

### Auxin Transport Inhibitors Induce Pseudonodules on Legumes Forming Indeterminate Nodules

Several studies have reported the ability of certain indeterminate nodule-forming legumes to form nodule-like structures, also termed pseudonodules, in response to ATIs. We wanted to investigate if the predisposition to form pseudonodules in response to ATIs is unequivocally confined to indeterminate-nodule forming legumes. To simulate the transient nature of the acropetal auxin transport inhibition in *M. truncatula*, we performed temporary flooding of selected legume species with the ATIs TIBA and NPA (Rightmyer and Long, 2011). We selected *M. truncatula* and *Trifolium subterraneum* to represent indeterminate nodule-forming legume species. On the other hand, *L. japonicus*, *Glycine max* (soybean), *Vicia faba* (broad bean), *Vigna unguiculata* (snake bean), and *M. atropurpureum* (siratro) were selected to represent determinate nodule-forming legume species. *Sesbania rostrata* can form indeterminate- or determinate-type nodules, depending on aeration status of the roots (Fernández-López et al., 1998). Selecting this species posed an interesting question as to whether ATIs could induce pseudonodules on this “dual nodule-type” legume.

**FIGURE 4:**
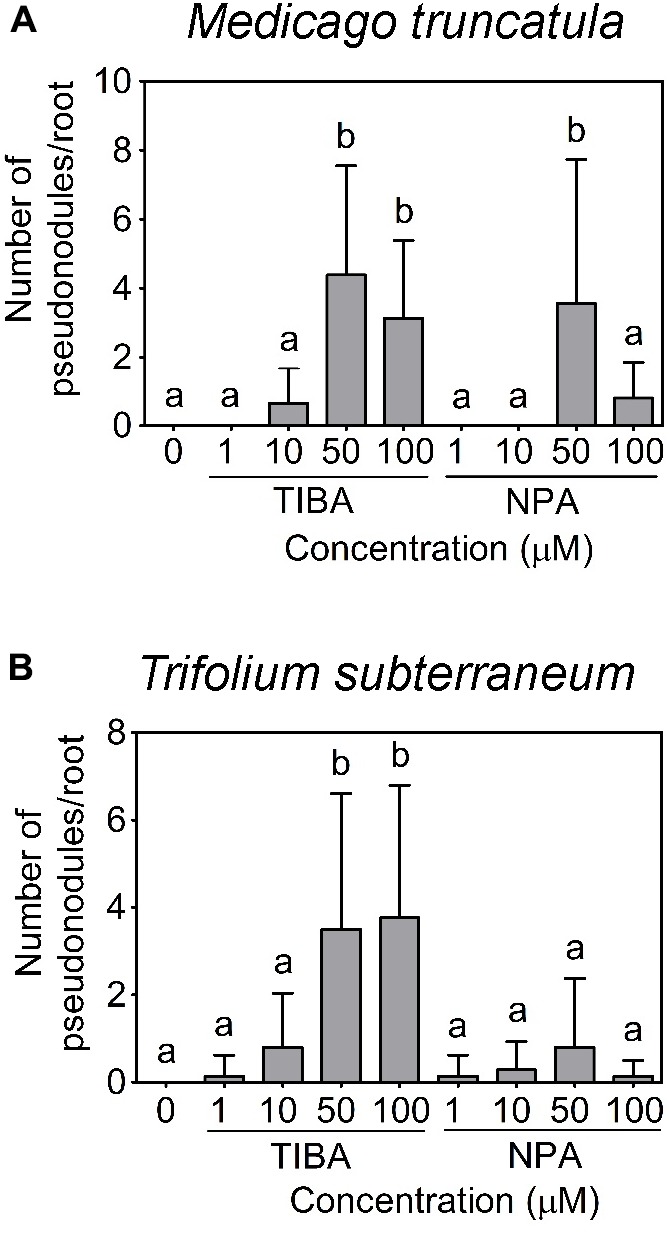
Pseudonodule formation in response to TIBA and NPA treatment at various concentrations. Pseudonodule formation on the roots of **(A)** *M. truncatula* and **(B)** *Trifolium subterraneum* (subclover), in response to TIBA and NPA treatments within the concentration range of 0–100 μM. A two-way ANOVA with a Dunn’s multiple comparison post-test were used for statistical analyses. Different lowercase letters indicate statistically different number of pseudonodules per root (*p* < 0.05, *n* = 20–25 per treatment). Graphs show mean and SD. Abbreviations: TIBA, 2,3,5-triiodobenzoic acid and NPA, 1-*N*-naphthylphthalamic acid.

Using a temporary flooding system, we found that both TIBA and NPA could induce pseudonodules on the roots of *M. truncatula* and *T. subterraneum*. The optimal concentration of TIBA and NPA to induce pseudonodules on *M. truncatula* was 50 μM (**Figure 4A**). At 100 μM, TIBA induced pseudonodules on *M. truncatula* roots at a similar frequency as 50 μM, but a similar concentration of NPA reduced pseudonodules formed on the roots of *M. truncatula* (**Figure 4A**). TIBA, but not NPA, could induce pseudonodule formation at 10 μM on *M. truncatula* roots (**Figure 4A**). For *T. subterraneum*, pseudonodules formed at the highest frequency at 50 and 100 μM TIBA treatment (**Figure 4B**). Pseudonodules were observed on the roots of *T. subterraneum* treated with 1 and 10 μM TIBA, as well as 1, 10, 50, and 100 μM NPA, although pseudonodule numbers were significantly lower (**Figure 4B**). In both legumes, TIBA overall induced significantly more pseudonodules than NPA at the concentrations tested (**Figures 4A,B**; two-way ANOVA, *p* < 0.001). We also tested primary root growth at various concentrations of TIBA and NPA on *M. truncatula* and *L. japonicus* to investigate if they had any adverse effects on root development. At concentrations up to 100 μM TIBA in *M. truncatula* (Supplementary Figure 1A) and 50 μM TIBA in *L. japonicus* (Supplementary Figure 1B), primary root growth was not significantly affected when compared with the control treatment. However, in both legume species, a 10-μM NPA treatment already significantly reduced primary root growth (Supplementary Figures 1A,B), suggesting a stronger pleiotropic effect caused by NPA treatment. We did not observe any pseudonodules forming on the roots of *L. japonicus*, *G. max*, *V. faba*, *V. unguiculata*, or *M. atropurpureum* in response to the temporary flooding with either TIBA or NPA (Supplementary Figure 2; 15–25 plants analyzed per species). Interestingly, a few pseudonodules formed on the roots of *S. rostrata* in response to TIBA or NPA treatment (10 out of 96 plants) at a range of concentrations tested (1, 10, 50, or 100 μM) under non-flooded conditions that lead to indeterminate nodule types (Fernández-López et al., 1998).

**FIGURE 5:**
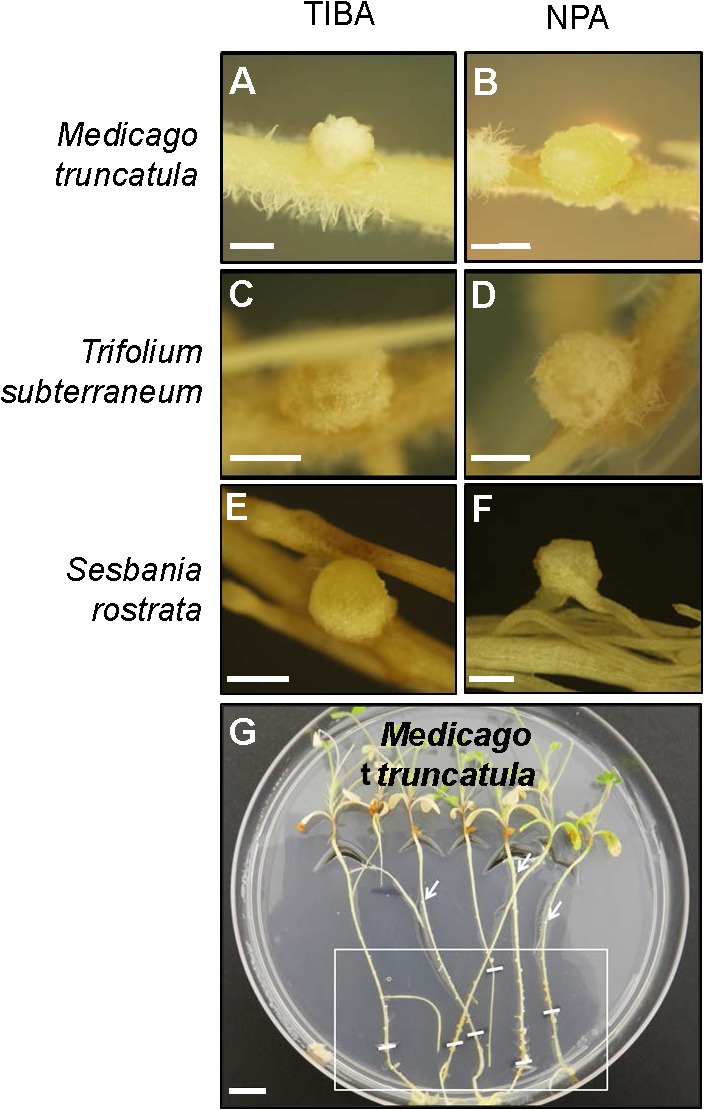
Pseudonodule formation on the roots of legumes in response to TIBA or NPA treatments. Pseudonodules formed on the roots of **(A,B)** *M. truncatula*, **(C,D)** *T. subterraneum* (subclover), and **(E,F)** *Sesbania rostrata*, in response to treatment with either 50 μM TIBA or 50 μM NPA. **(G)** Pseudonodules were formed along the entire roots of *M. truncatula*. The area on the roots encapsulated by the white box indicates the region where the majority of pseudonodules were found on each root. White lines within the box indicate locations of the root tip when the roots were first subjected to TIBA treatment. White arrows indicate pseudonodules found at older parts of the roots. Scale bars represent 1 mm in **(A–F)** and 1 cm in **(G)**. Abbreviations: TIBA, 2,3,5-triiodobenzoic acid and NPA, 1-*N*-naphthylphthalamic acid.

**FIGURE 6:**
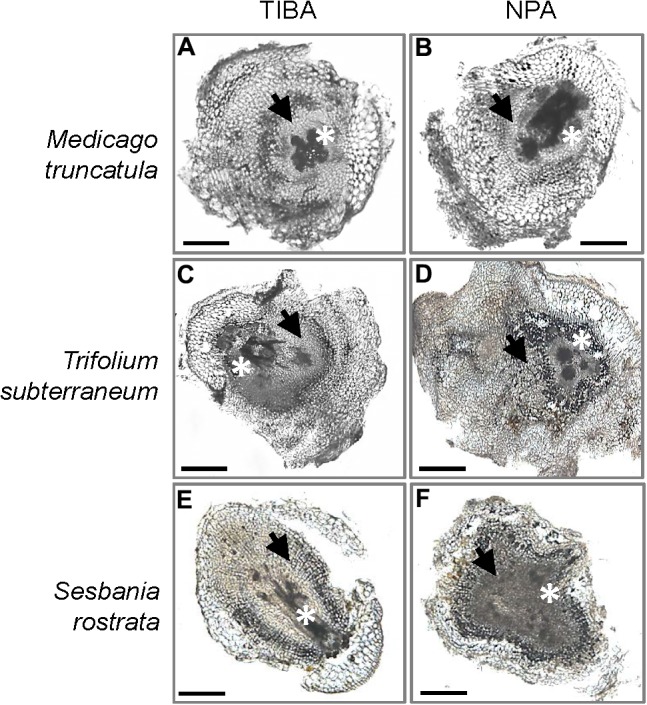
Cross sections of pseudonodules induced by TIBA or NPA treatment. Pseudonodules were induced on the roots of **(A,B)** *M. truncatula*, **(C,D)** *T. subterraneum* (subclover), and **(E,F)** *S. rostrata*. Asterisks indicate location of the primary root vasculature. Arrows indicate pericycle and endodermal cell divisions. Scale bars represent 500 μm. Abbreviations: TIBA, 2,3,5-triiodobenzoic acid and NPA, 1-*N*-naphthylphthalamic acid.

The outward appearance of pseudonodules on *M. truncatula* (**Figures 5A,B**), *T. subterraneum* (**Figures 5C,D**), and *S. rostrata* (**Figures 5E,F**) resembled rhizobia-induced nodules. Interestingly, although pseudonodules on *M. truncatula* primarily formed on younger parts of the roots (where rhizobia-induced nodules form), they could also be found on more mature parts of the roots (**Figure 5G**). Cross sections of these structures revealed a more diffuse and randomized nature of cell divisions, as opposed to a more controlled pattern of cell divisions observed in rhizobia-induced nodules. Mature pseudonodules formed on *M. truncatula* (**Figures 6A,B**), *T. subterraneum* (**Figures 6C,D**), and *S. rostrata* (**Figures 6E,F**) all displayed cell divisions in the pericycle, endodermis, and cortex (**Figure 6**). However, pseudonodules were characterized by more extensive pericycle and endodermal cell divisions, rather than the predominantly cortical cell divisions observed in rhizobia-induced nodules.

The legume *S. rostrata* can also form nodules on its stems. We did not observe pseudonodules forming on the stems of *S. rostrata* in response to TIBA or NPA treatment. However, unlike the adventitious root buds found on stems under control treatment (Supplementary Figures 3A,B), we found a higher occurrence of adventitious structures resembling bumps forming on the stems (Supplementary Figures 3D,E,G,H). Cross sections of these structures revealed a thicker layer of tissue with high cell wall autofluorescence (Supplementary Figures 3F,I), visualized under UV light, in comparison to the control (Supplementary Figure 3C).

Previously, we demonstrated that the formation of pseudonodules involved an inhibition of acropetal auxin transport in *M. truncatula* roots (Ng et al., 2015). Thus, we also tested auxin transport regulation in response to TIBA treatment (50 μM) at 6, 24, and 48 h.p.i., similar to the measurements performed for rhizobia inoculation. Unlike the transient nature of acropetal auxin transport inhibition in *M. truncatula* (**Figures 1C**, **2A**), acropetal auxin transport decreased significantly in response to TIBA treatment at 6, 24, and 48 h.p.i. (**Figure 2B**). Although rhizobia inoculation failed to reduce acropetal auxin transport in *L. japonicus*, TIBA treatment also significantly reduced acropetal auxin transport in this legume (**Figure 3B**).

### Changes in Auxin Response and Concentration during Nodule Formation in *Medicago truncatula* and *Lotus japonicus*

**FIGURE 7:**
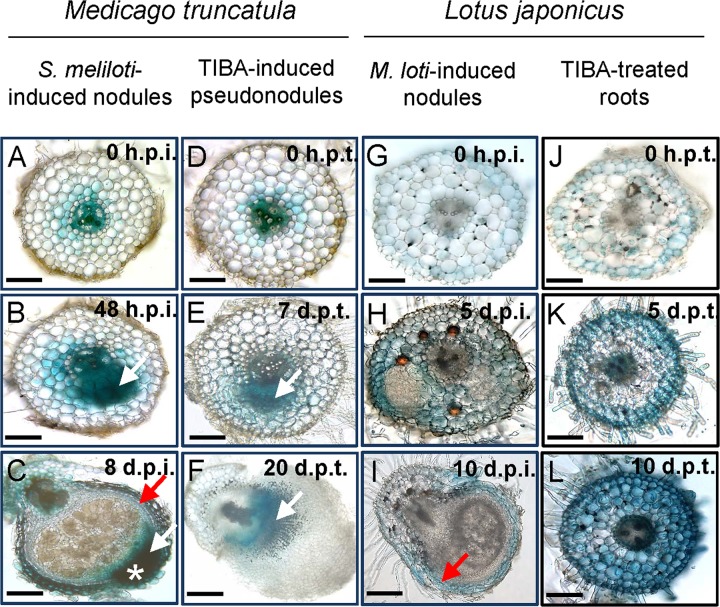
Auxin response as seen by *GH3:GUS* expression in *M. truncatula* and *L. japonicus* roots or nodules. Auxin response in different tissues in **(A–C)** *S. meliloti*-induced nodules on *M*. *truncatula* roots; **(D–F)** TIBA-induced pseudonodules on *M*. *truncatula* roots; **(G–I)** *M. loti*-induced nodules on *L*. *japonicus* roots; and **(J–L)** TIBA-treated roots of *L*. *japonicus*. The developmental stages are indicated by the time after *S. meliloti*/TIBA/*M*. *loti* treatment in each case. White arrows indicate auxin response in dividing cells. Red arrow indicates auxin response in the nodule vasculature. White asterisk indicates the location of the nodule meristem, where an enhanced auxin response is observed. Scale bars represent 200 μm. Abbreviations: h.p.i., hours post-inoculation; d.p.i., days post-inoculation; h.p.t., hours post-treatment; and d.p.t., days post-treatment.

We localized auxin responses in both *M. truncatula* and *L. japonicus* fully transformed plants carrying the *GH3:GUS* reporter (Pacios-Bras et al., 2003; van Noorden et al., 2007). In *M. truncatula*, auxin responses were enhanced in early nodule primordia at 48 h.p.i. in divided pericycle, endodermis, and inner cortex cells (**Figure 7B** and Supplementary Figure 4). In mature nodules, an auxin response was found in the nodule vasculature and the nodule meristem (**Figure 7C**).

During pseudonodule formation in *M. truncatula*, the first small nodule primordia were found at 7 days following TIBA application, which was slower than formation of equivalent nodule primordia at 48 h after rhizobia inoculation. We found no enhanced *GH3::GUS* expression in the cortex at 48 h after TIBA treatment (Supplementary Figure 5). An increased auxin response was localized in the dividing pericycle, endodermis, and inner cortex cells at the early primordium stage at 7 days after TIBA application, similar to rhizobia-induced nodule primordia (**Figure 7E**). However, in more mature pseudonodules (20 days post-treatment), *GH3::GUS* expression did not resemble that of rhizobia-induced nodules and was confined to pericycle, endodermal, and inner cortical cell layers (**Figure 7F**).

In *L*. *japonicus*, induction of auxin responses occurred in cortical cells, mainly those surrounding early nodule primordia or those that appeared to have just divided, while primordia themselves showed very low *GH3::GUS* expression (**Figure 7H** and Supplementary Figure 4). We did not detect any changes in auxin response before the onset of cell divisions (**Figures 7A,D,G,J**). In mature *L*. *japonicus* nodules, auxin responses were restricted to the vasculature (**Figure 7I**). Nodules in *L. japonicus* do not retain an apical meristem, and no apical *GH3::GUS* response was seen. In *L. japonicus* roots treated with TIBA, we detected increased *GH3::GUS* responses first in outer cortical and epidermal cells (5 days post-treatment) and later in the whole root (10 days post-treatment), but despite serial sectioning of multiple roots, we never detected any divided cells in the cortex (**Figures 7K,L**).

In parallel to the auxin responses, we quantified auxin content in inoculated root segments encompassing the inoculation site. We previously reported increased IAA content in inoculated *M. truncatula* root segments at 24 h.p.i., but not at 6 h.p.i. (Ng et al., 2015). Measurements at 48 h.p.i. in this study showed no significant changes in IAA content (Supplementary Figure 6). However, we found a significant decrease in the concentration of IAA-Ala at 48 h.p.i. that was not found at either 6 or 24 h.p.i. (Ng et al., 2015; Supplementary Figure 6). The auxins PAA, 4-Cl-IAA, and IAA-Val were not detected in any of the samples measured.

**FIGURE 8:**
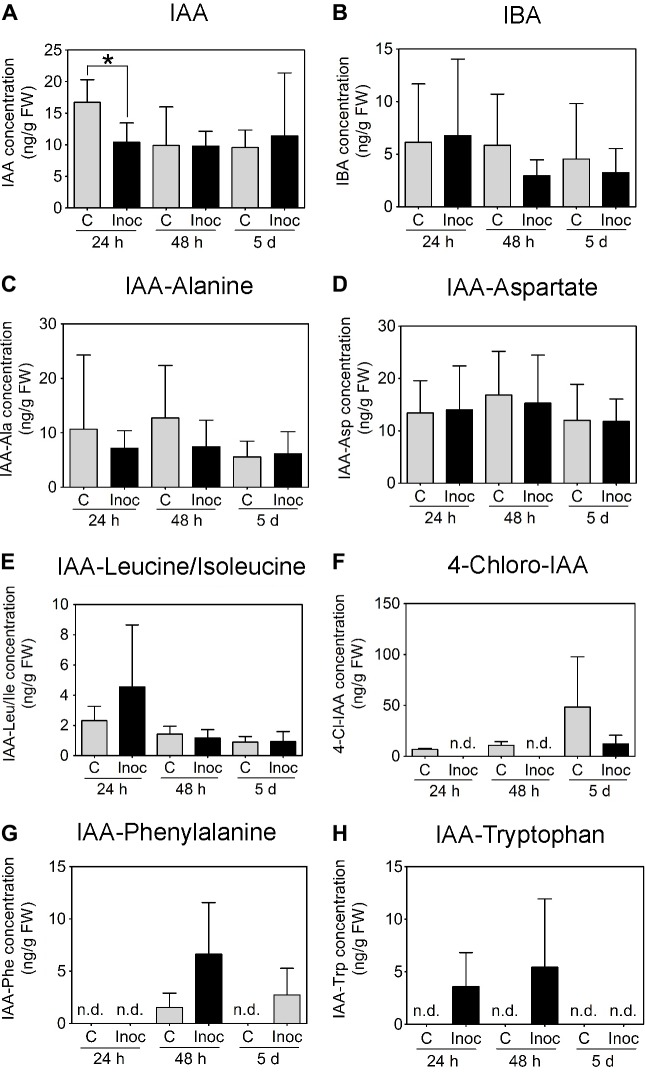
Auxin content in developing *L. japonicus* nodules (4 mm long segments around the inoculation site). Endogenous auxin concentration (ng g^-1^ FW) in *L. japonicus* roots inoculated with *M. loti*- or mock-treated roots was measured at 24 h, 48 h, and 5 days post-inoculation. Auxins detected in *L. japonicus* roots include **(A)** IAA, **(B)** IBA, **(C)** IAA-Ala, **(D)** IAA-Asp, and **(E)** IAA-Leu/Ile. **(F)** 4-Cl-IAA, **(G)** IAA-Phe, and **(H)** IAA-Trp were not consistently detected at all time points. A two-way ANOVA and a Student’s *t*-test were used for statistical analyses (*p* < 0.05; *n* = 3–6 per treatment or time point). Asterisk in **(A)** indicates a significant difference in auxin concentration (*p* < 0.05). Graphs show mean and SD. Abbreviations: FW, fresh weight; C, control; Inoc, inoculated; n.d., not detected; IAA, indole-3-acetic acid; and IBA, indole-3-butyric acid.

In contrast to *M. truncatula*, we measured a temporary decrease in IAA concentration in *L. japonicus* root segments encompassing the nodulation zone at 24 h.p.i. (**Figure 8A**). No IAA changes were detected between control and inoculated *L. japonicus* roots at 48 h.p.i. or 5 days post-inoculation (**Figure 8A**). The concentrations of IBA, IAA-Alanine, IAA-Aspartate, and IAA-Leucine/Isoleucine were not altered by rhizobia inoculation (**Figures 8B–E**). The auxins 4-chloro-IAA, IAA-Phenylalanine, and IAA-Tryptophan were not consistently detected in the root segments (**Figures 8F–H**).

## Discussion

In our study, we posed two main questions:

(1) Is acropetal auxin transport inhibition during nodulation restricted to indeterminate nodules, and what are possible reasons for this?
(2) Which other mechanisms could contribute to increased auxin responses in the cortex during nodule initiation?

### Is Acropetal Auxin Transport Inhibition during Nodulation Restricted to Indeterminate Nodulation, and What Are Possible Reasons for This?

Consistent with previous results in *M. truncatula*, we observed a transient reduction in acropetal auxin transport following rhizobia treatment at 24 h.p.i., coinciding with the very earliest cell divisions (Wasson et al., 2006; van Noorden et al., 2006; Plet et al., 2011; Ng et al., 2015). For *L. japonicus*, Pacios-Bras et al. (2003) previously demonstrated an increase in acropetal auxin transport capacity in response to Nod factor treatment at 48 h.p.i., although those experiments were not statistically analyzed. Here, we included two additional features to our measurements: (1) we included additional time points to assess if this increase in auxin transport capacity was of a transient or prolonged nature and correlated with the onset of cell divisions and (2) we measured auxin transport in equivalent segments below the inoculation spot. Crucially, this study provides a direct comparison of two corresponding segments in *M. truncatula* and *L. japonicus*. Unlike the short-term nature of acropetal auxin transport inhibition during indeterminate nodule initiation, we found that an increased auxin transport capacity in *L. japonicus* started at 12 h.p.i., i.e., before the very earliest cortical cell divisions, and continued at 48, 72, 96, and 120 h.p.i., where cell divisions were more extensive.

In *M. truncatula*, knockdown of Mt*PIN4* (ortholog of At*PIN1* mediating acropetal auxin transport; Schnabel and Frugoli, 2004; Kohlen et al., 2017) reduced nodulation (Huo et al., 2006), and transcription of this gene was one of the most highly upregulated by Nod factor treatment (Plet et al., 2011), suggesting that acropetal auxin transport regulation might occur through MtPIN4, although it may involve other auxin transporters. So far it is unknown how the increased acropetal auxin transport in *L. japonicus* is regulated. It would be essential to quantify *PIN* gene expression in *L. japonicus* in the future, and analyze mutants of auxin transporters.

The lack of auxin transport inhibition during *L. japonicus* nodule development was supported by the inability of TIBA or NPA to induce pseudonodules in this species. We observed pseudonodule formation on *M. truncatula*, subclover, and *S. rostrata*, and pseudonodules were also reported in alfalfa and white sweet clover (Hirsch et al., 1989; Wu et al., 1996). Although *S. rostrata* has the capacity to form both nodule types, it typically forms indeterminate-type nodules under non-waterlogging and well-aerated growth conditions (Fernández-López et al., 1998), and our growth system and ATI assays simulated these conditions. Lack of pseudonodule formation on *L. japonicus* in response to ATIs had previously been observed (Takanashi et al., 2011), even though TIBA treatment reduced acropetal auxin transport capacity in *L. japonicus* roots similar to *M. truncatula*. Curiously, gibberellins were able to induce pseudonodules on *L. japonicus*, but not alfalfa roots (Kawaguchi et al., 1996). The mode of action or physiological effects of gibberellins were, however, not examined. Nevertheless, gibberellins are not likely to inhibit acropetal auxin transport because of their failure to induce pseudonodules on alfalfa. In addition to *L. japonicus*, pseudonodules were not found on any of the other examined legume species forming determinate nodules, including *G. max*, *V. faba*, *V. unguiculata*, or *M. atropurpureum.* Pseudonodules had been reported as “few” in response to TIBA or NPA in *M. atropurpureum* (Relić et al., 1993), but no photos were shown in that study and we could not confirm this finding.

Pseudonodules formed in *M. truncatula* in response to TIBA showed *GH3::GUS* expression in the base, but no expression at the pseudonodule “tip,” where rhizobia-induced nodules showed strong *GH3::GUS* expression in the nodule meristem. This suggests the lack of a pseudonodule meristem.

Why would ATIs not induce pseudonodules in determinate nodule-forming species? *M. truncatula* roots treated with TIBA formed pseudonodules, and at the earliest stages of their formation, these pseudonodules showed high auxin (*GH3::GUS*) responses in the pericycle, endodermis, and inner cortex, similar to indeterminate nodule primordia. Modeling has shown that acropetal auxin transport inhibition would produce this pattern of auxin accumulation (Deinum et al., 2012). When we treated *L. japonicus* roots with TIBA, we found increased *GH3::GUS* expression in a different pattern – in the outer cortex, and later on in all cortex cells – but no small primordia were formed. Thus, it is likely that the difference in the tissue types involved in the early cell divisions (pericycle, endodermis, and inner cortex in indeterminate, and outer cortex for determinate nodules) dictates whether auxin transport changes contribute to nodule initiation. It is also possible that cortical responses to auxin differ in indeterminate and determinate nodule-forming species, for example in sensitivity, and this will have to be further explored in the future. For instance, it might be possible that auxin concentrations generated in the cortex in response to ATIs become super-optimal for determinate nodules (Turner et al., 2013).

### Which Other Mechanisms Could Contribute to Increased Auxin Responses in the Cortex during Nodule Initiation?

During the earliest stage of nodule initiation, both *M. truncatula* and *L. japonicus* showed increased auxin (*GH3::GUS*) response in the inner or outer/middle cortex, respectively. It is possible that the increased auxin response seen in dividing outer cortical cells in *L. japonicus* is in response to altered sensitivity to auxin. Studies in soybean have demonstrated the role of a number of microRNAs controlling the expression of auxin receptors and response genes that could change sensitivity toward auxin (Mao et al., 2013; Turner et al., 2013). Another mechanism to explain the initial increased auxin response could include auxin synthesis in the cortex, and evidence for the induction of auxin synthesis genes in *L. japonicus* was found previously (Suzaki et al., 2012). However, we did not detect any significant increase in auxin concentration in that area in *L. japonicus* roots. Intriguingly, a reduction in IAA content was measured at 24 h.p.i., although we did not detect any changes in auxin transport or auxin response at that stage. Currently, we cannot explain the significance of this finding; it could be due to increased auxin degradation. In *M. truncatula*, the increased auxin response at the early primordium stage at 48 h.p.i. was not accompanied by increased auxin concentrations, either. This suggests that it is either difficult to detect increased auxin concentrations in small numbers of cells, as 4 mm root segments were harvested for this assay, or that there is no linear relationship between *GH3::GUS* expression and auxin concentration.

Genetic studies on the role of auxin transport carriers during nodulation in *L. japonicus* are lacking. One report highlighted the role of an ABCB-type multidrug resistance protein, LjABCB1, in mature *L. japonicus* nodules, but did not examine its role during early nodule development. The authors proposed that LjABCB1 exports auxin toward infected cells in the nodule, based on the selective localization of the protein at adjacent uninfected cells (Takanashi et al., 2012). Future studies will clearly have to unravel the regulation of various auxin transporters in *L. japonicus* (Kohlen et al., 2017). In addition, it will be necessary to model the changes in acropetal auxin transport in *L. japonicus* computationally, to find out if these observed increases in auxin transport would account for the increased auxin responses in the cortex. Previous modeling has shown that auxin accumulation in the middle/outer cortex could be explained by radial repositioning of PIN proteins (Deinum et al., 2012, 2016).

Apart from the differences between *M. truncatula* and *L. japonicus* in auxin transport at the time of the first cortical cell divisions, we also found differences in auxin responses at the primordia stage. While auxin responses remained high in young nodule primordia of *M. truncatula*, auxin response diminished quickly in *L. japonicus* nodule primordia as reported by other studies (Mathesius et al., 1998; Takanashi et al., 2011; Suzaki et al., 2012; Turner et al., 2013). It is possible that changes in auxin sensitivity in the cortex differentiate the conditions suitable for nodule development in species forming indeterminate and determinate nodules: While reduced auxin sensitivity increased nodule numbers in soybean (Nizampatnam et al., 2015; Wang et al., 2015), a window of higher auxin sensitivity may be required for indeterminate nodule initiation (Mao et al., 2013). However, it was also demonstrated that silencing of the auxin receptor TIR1 in soybean reduced nodulation, suggesting that auxin responses are necessary for determinate nodule formation (Cai et al., 2017). Thus, correct auxin responses in space and time are crucial for nodule development.

In summary, we found no evidence that inhibition of acropetal auxin transport occurs during nodule initiation in *L. japonicus*, and it is unlikely to be a mechanism that creates the correct auxin gradients for other legume species forming determinate nodules because ATIs did not induce any cortical cell divisions in a number of tested species, in contrast to species forming indeterminate nodules. While increased auxin responses occurred in the earliest dividing cortex cells of *L. japonicus*, no significant increase in auxin concentration was measured. In contrast, *M. truncatula* showed more sustained auxin responses in nodule primordia, although increased auxin concentration was only detected at 24 h, preceding nodule primordia. Thus, it is possible that changes in auxin sensitivity in cortical cells, without measurable changes in auxin content, contribute to increased auxin responses.

## Materials and Methods

### Plant Material and Growth Conditions

Plant species used in this study included *M. truncatula* wild-type cultivar Jemalong A17 (South Australian Research and Development Institute, Adelaide, Australia), *L. japonicus* ecotype Gifu B-129 (Biological Resource Center in *Lotus* and *Glycine*, University of Miyazaki, Japan), *T. subterraneum* cv. Karridale (Clean Seeds, Bungendore, Australia), *G. max* cv. Bragg (Prof. Peter Gresshoff, University of Queensland), *S. rostrata* (Prof. Sofie Goormachtig, Ghent University, Belgium), *M. atropurpureum* (Selected Seeds, Pittsworth, Australia), *V. unguiculate* and *V. faba* cv. Coles Early Dwarf (both Mr. Fothergill’s Seeds Pty Ltd., South Windsor, Australia).

Seeds of *M. truncatula, L. japonicus*, *T. subterraneum*, and *M*. *atropurpureum* were scarified with sand paper, surface-sterilized in 6% (w/v) sodium hypochlorite for 10 min, then washed with sterilized milliQ water five times. Next, *M*. *truncatula* and *L*. *japonicus* seeds were imbibed in sterilized milliQ water containing 0.25 mg ml^-1^ of the antibiotic augmentin for 6 h with gentle mixing by rotation to reduce bacterial contamination. Sterilized seeds were washed once with sterilized milliQ water and then plated on Fahråeus (F) medium (Fahraeus, 1957) for *M*. *truncatula* or 1/4; strength Broughton and Dilworth medium (Broughton and Dilworth, 1971) for *L*. *japonicus*. For *T. subterraneum* and *M*. *atropurpureum*, seeds were sown straight onto F plates without the augmentin incubation step. Seeds were incubated at 4°C in the dark for 48 h. Germination of seeds was synchronized by incubating the plates at 25°C for 24–48 h with plates inverted. Seedlings with radicle length of approximately 5–10 mm were transferred onto F plates (*M*. *truncatula*, *T*. *subterraneum*, and *M*. *atropurpureum*) or 1/4; B&D plates (*L*. *japonicus*). Plates were semi-sealed with Parafilm, placed vertically in a container with a black cardboard interspersed between each plate to shield roots from direct light. Plates were incubated at 25 (*M*. *truncatula*, *T*. *subterraneum*, and *M*. *atropurpureum*) or 20°C (*L*. *japonicus*), with a 16 h light and 8 h dark period at 150 μmol m^-2^ s^-1^ light intensity.

For soybean, the germination protocol was based on Brechenmacher et al. (2009). Seeds were soaked in 0.1 N HCl for 10 min and then washed with tap water five times. Seeds were then surfaced-sterilized with 6% (w/v) sodium hypochlorite for 15 min and then washed with sterilized milliQ water five times, air dried for 20 min, and subsequently sown on 1/4; B&D plates. Seeds were left at 25°C in the dark for germination over several days. Germinated soybeans were transferred into pots containing autoclaved vermiculite (Grade 3), watered with 1/4; B&D medium, and grown at 16 h light and 8 h dark period at 150 μmol m^-2^ s^-1^ light intensity in a glasshouse.

For *S. rostrata*, the germination protocol was modified from Goethals et al. (1989). Seeds were immersed in 4 M sulfuric acid for 1 h and washed five times with tap water. This was followed by surface sterilization with 6% (w/v) sodium hypochlorite for 15 min and then five washes with sterilized milliQ water. Sterilized seeds were imbibed overnight in sterilized milliQ water. Imbibed seeds were plated onto F plates and left at 25°C in the dark for germination over several days. Germinated seedlings were transferred into glasshouse pots containing autoclaved vermiculite (Grade 3) and watered with F medium twice a week.

Seeds of *V. unguiculata* and *V. faba* were surface-sterilized with 6% (w/v) sodium hypochlorite for 15 min and then washed with sterilized milliQ water five times. Seeds were directly planted into pots containing autoclaved vermiculite (Grade 3) and thinned to one plant per pot after germination. Plants were watered with F medium twice a week and grown in a glasshouse.

### Bacterial Strains and Inoculation Conditions

The *S. meliloti* strain A2102, a triple nod mutant for nodD1, nodD2, and nodD3 derived from the WT strain Sm1021, containing the pE65 plasmid encoding a constitutively overexpressed copy of nodD3 (Barnett et al., 2004) was used for all inoculations on *M*. *truncatula* (kindly provided by Dr. Melanie Barnett and Prof. Sharon Long, Stanford University, Stanford, CA, United States), hereafter referred to as “E65.” This strain was used to compare results from this study to that of Ng et al. (2015). We previously established that the E65 strain (Ng et al., 2015) inhibited auxin transport similarly to the wild-type strain 1021 (Wasson et al., 2006). The E65 strain was maintained on Bergensen’s Modified Medium (BMM) (Rolfe and Gresshoff, 1988) supplemented with 10 μg ml^-1^ tetracycline and 100 μg ml^-1^ streptomycin (Sigma Chemicals). For *L. japonicus*, inoculation (OD_600__nm_ = 0.05) was performed with *Mesorhizobium loti* strain MAFF303099, maintained on Tryptone-Yeast (TY) medium.

For inoculation of *M. truncatula*, an overnight culture of *S. meliloti* strain E65 in BMM at 28°C was used. The optical density (OD_600__nm_) of the culture was adjusted to 0.1 for spot-inoculation. Spot-inoculation was performed by placing ∼1 μl of *S. meliloti* culture or BMM, onto the root surface 2 mm above the root tip, corresponding to the nodulation-susceptible zone (Bhuvaneswari et al., 1981). For analyzing *GH3::GUS* expression, spot-inoculation was performed with a glass capillary pulled into a fine tip over a flame and glued to a hypodermic needle.

For spot-inoculation of *L*. *japonicus*, a liquid culture of *M*. *loti* in TY medium was incubated for 3 days at 28°C. The OD_600__nm_ of the cultures was adjusted to 0.05. Spot-inoculation was performed by placing ∼1 μl of rhizobia culture or TY as a negative control, 2 mm above the root tip.

### Flood Treatment with Auxin Transport Inhibitors

Seeds were germinated as described above. Seedlings of *M*. *truncatula*, *L. japonicus*, *T*. *subterraneum*, and *M. atropurpureum* were treated while growing on agar plates. At 1 week post-transfer onto agar medium, seedlings were treated by flooding, as described in Rightmyer and Long (2011). Diluted solutions of NPA and TIBA (Sigma Chemicals) were made in sterile 50 ml Falcon tubes. Control treatments contained equivalent dilution of methanol (which was used as a solvent for stock solutions). The concentrations of ATIs were chosen because they were previously demonstrated to induce pseudonodules on *M. truncatula* (Rightmyer and Long, 2011). Seedlings were flooded with 20–30 ml of diluted ATIs for 10 s, and then the solution was decanted. Pseudonodules were analyzed 3 weeks after treatment with ATIs.

For plants grown in pots (*G. max*, *S. rostrata*, *V. faba*, *V. unguiculata*, and *M. truncatula* as a positive control for pot experiments), 50 ml of ATI diluted in growth medium was applied to each pot. This was repeated after 1 week and plants were then left to grow for another 3 weeks before uprooting the plants and analyzing for pseudonodule formation.

### Auxin Quantification

Auxin quantification was based on Ng et al. (2015). Commercial auxin standards and a deuterated internal standard were used to determine elution times, collision energies, detection limits, and for absolute quantification. Auxin standards were obtained from OlChemim (IAA-Phenylalanine, IAA-Leucine, IAA-Valine, IAA-Tryptophan, 4-Cl-IAA), Sigma (IAA-Aspartate, IAA-Alanine, IAA-Isoleucine, IAA, IBA, PAA) and Cambridge Isotope Laboratories (indole-2,4,5,6,7-d5-3-acetic acid).

Plant roots were collected at 6, 24, and 48 h.p.i. with rhizobia. Root segments of 4 mm spanning the spot-inoculation site were collected and snap-frozen immediately in liquid nitrogen. A total of 30–40 root segments were collected for each treatment, for each biological replicate. Between 5 and 10 biological replicates were analyzed for each time point and species. The frozen tissue samples were mechanically lysed with stainless steel beads in a Qiagen TissueLyser LT with a pre-cooled tube holder. To each tube 20 μl of the internal standard (1 μg ml^-1^ of 3-[^2^H_5_]indolylacetic acid) followed by 500 μl extraction solvent (methanol:propanol:glacial acetic acid, 20:79:1, v/v/v) were added and auxin extraction was performed in a sonicator bath for 30 min at 4°C. Samples were then centrifuged at 16,000 × *g* for 15 min. The supernatant was transferred to a fresh tube and subsequently dried in a Speedvac centrifuge. Extraction was repeated once, and the supernatant combined with the (dried) supernatant from the first extraction, and subsequently evaporated in a Speedvac centrifuge. Vacuum-dried samples were resuspended with 100% methanol, vortexed for 5 s, and filtered through a Nanosep MF GHP 0.45 μm filter (Pall Life Sciences) by centrifugation at 16,000 × *g* for 1 min. The resuspension step was repeated once. The eluent containing the auxin extracts was transferred to an amber vial and vacuum-dried. Samples were stored at -80°C until analysis. Prior to analysis, samples were taken out from the freezer to equilibrate to room temperature. Each sample was resuspended in 50 μl methanol (Acros Organics):water (60:40, v/v).

Tandem mass spectrometry was performed using an Agilent 6530 Accurate Mass LC–MS Q-TOF (Santa Clara, CA, United States). Samples were subjected to ESI in the Jet Stream interface in both ion positive and negative polarities. Based on optimized LC-ESI-Q-TOF parameters using auxin standards, the auxins IAA, IBA, and IAA-Ala had better sensitivity in the positive mode. The other auxin species were better detected in the negative mode. Optimized conditions in the positive mode were as follows: gas temperature 250°C, drying gas 5 l min^-1^, nebulizer 30 psig, sheath gas temperature 350°C and flow rate of 11 l min^-1^, capillary voltage 2500 V, nozzle voltage 500 V, and fragmentor voltage 138 V. Conditions in the negative mode were as follows: gas temperature 300°C, drying gas 9 l min^-1^, nebulizer 25 psig, sheath gas temperature 350°C and flow rate of 11 l min^-1^, capillary voltage 3000 V, nozzle voltage 500 V, and fragmentor voltage 140 V. Samples were injected (7 μl) onto an Agilent Zorbax Eclipse 1.8 μm XDB-C18 2.1 × 50 mm column. Solvent A consisted of 0.1% aqueous formic acid and solvent B, 90% methanol/water with 0.1% formic acid. Free auxins and conjugates were eluted with a linear gradient from 10 to 50% solvent B over 8 min, 50–70% solvent B from 8 to 12 min (then held at 70% from 12 to 20 min) at a flow rate of 200 μl min^-1^. The quadrupole time-of-flight (Q-TOF) was run in targeted MS/MS mode using collision-induced dissociation (CID; N_2_ collision gas supplied at 18 psi with *m/z* 1.3 isolation window) where the MS extended dynamic range (2 Hz) was *m/z* 100–1000 with an acquisition rate of 3 spectra s^-1^ and MS/MS at *m/z* 50–1000 at 3 spectra s^-1^. Data were analyzed using Agilent Technologies MassHunter software (ver. B.5.0).

### Histochemistry and Microscopy

Beta-glucuronidase (GUS) staining was performed as described in van Noorden et al. (2007). Sections of 100 mm thickness were made using a Vibratome 1000 (Vibratome Company, St. Louis, MO, United States) and viewed under bright-field optics using a DMLB microscope (Leica Microsystems, Wetzlar, Germany), and images were collected with a mounted CCD camera (RT Slider; Diagnostic Instruments, Sterling Heights, MI, United States).

### Auxin Transport Studies

Tritium-labeled indole-3-acetic acid (^3^H-IAA) solution (7.5 μl of 1 mCi ml^-1^) (American Radiolabeled Chemicals, St. Louis, MO, United States) was diluted in 20 μl ethanol and mixed with 1.5 ml of melted and cooled 1% agarose at pH 4.8, in a 3 cm diameter Petri dish. The pH was chosen as it is close to the isoelectric point of IAA. Small blocks with dimensions of 2 mm × 2 mm × 2 mm were cut with a scalpel. This standardized the amount of ^3^H-IAA supplied to plants. Seedlings were pre-treated with rhizobia/ATIs prior to auxin transport study, as described below.

Acropetal auxin transport was performed in relation to the spot-inoculation site. Roots were cut 8 mm from the inoculation spot in the shootward direction (∼12 mm from the root tip). The shoot-containing segment was discarded, and a small ^3^H-IAA block (2 mm × 2 mm × 2 mm) placed on the cut end of the root-tip containing segment. A Parafilm strip was placed underneath the root segments to prevent diffusion of ^3^H-IAA from the growth media directly into parts of the root. Samples were incubated vertically for 6 h (*M. truncatula*) or 12 h (*L. japonicus*) in the dark to allow ^3^H-IAA to diffuse from the agar block through the cut end. The first 4 mm segment touching the ^3^H-IAA agar block was discarded. The root segments located just below the inoculation site were transferred into individual scintillation vials containing 2 ml scintillation fluid (Perkin-Elmer).

Samples were incubated on an INOVA 2100 Platform Shaker (New Brunswick Scientific) overnight at room temperature. Radioactivity was measured in a scintillation counter (Tri-Carb^®^ Liquid Scintillation Analyzer B2810TR, Perkin-Elmer) over 1 min each. The default settings for tritium decay measurement were used. A vial containing just the scintillation fluid was used as a blank for background subtraction during analysis.

### Statistical Analyses

Statistical analyses were carried out with Genstat 15th Edition (VSN International, Hemel Hempstead, United Kingdom) and Prism version 5.02.

## Author Contributions

JN and UM: conceived, acquired data, analyzed data, and drafted the work.

## Conflict of Interest Statement

The authors declare that the research was conducted in the absence of any commercial or financial relationships that could be construed as a potential conflict of interest.
