# Supplementary figures and images for: Acropetal Auxin Transport Inhibition Is Involved in Indeterminate But Not Determinate Nodule Formation
Source: Front Plant Sci. 2018 Feb 15;9:169. doi: 10.3389/fpls.2018.00169 (PMC5818462; doi:10.3389/fpls.2018.00169)

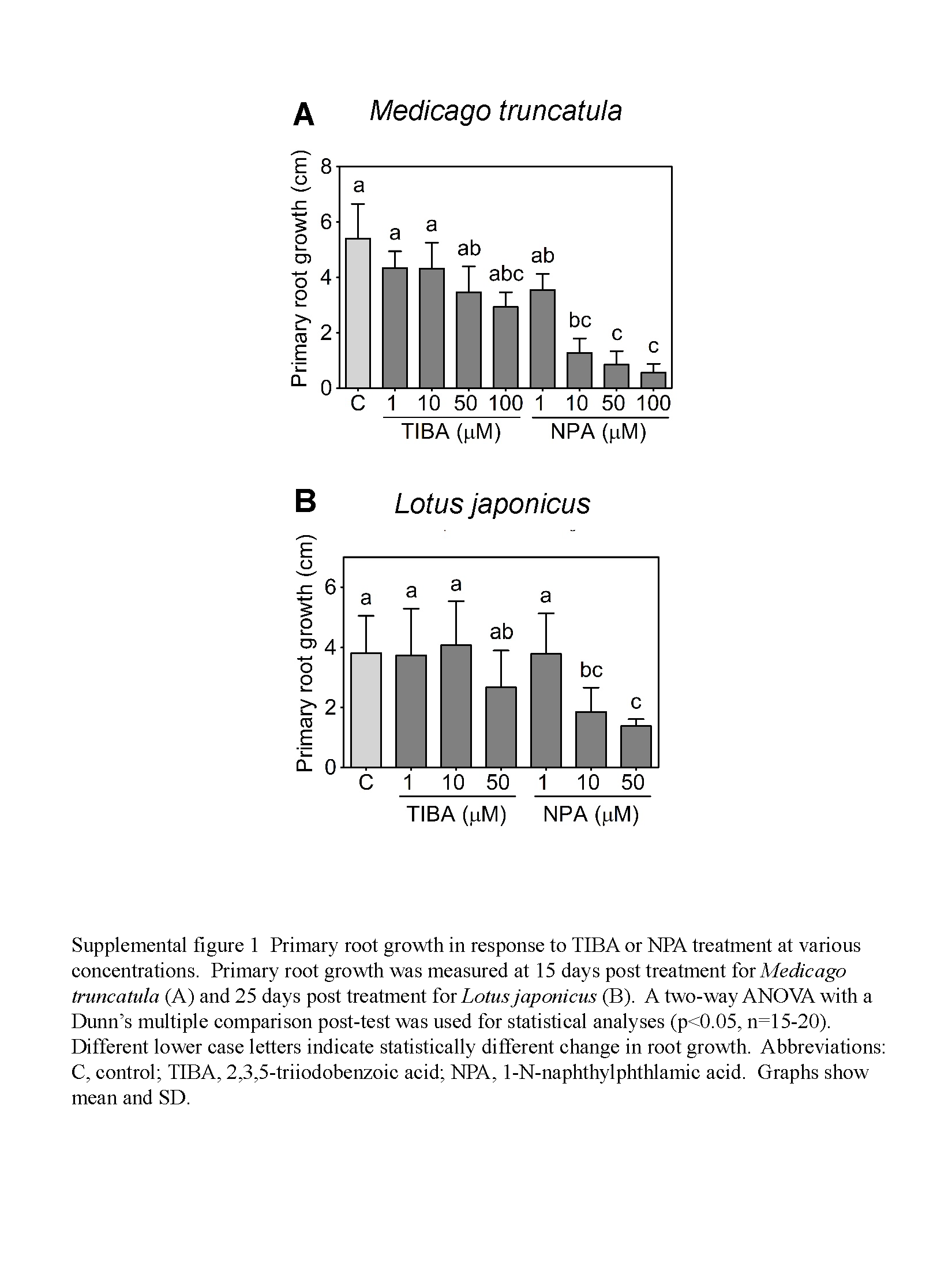

Supplement: Supplementary file 1 [file Image_1.TIFF]

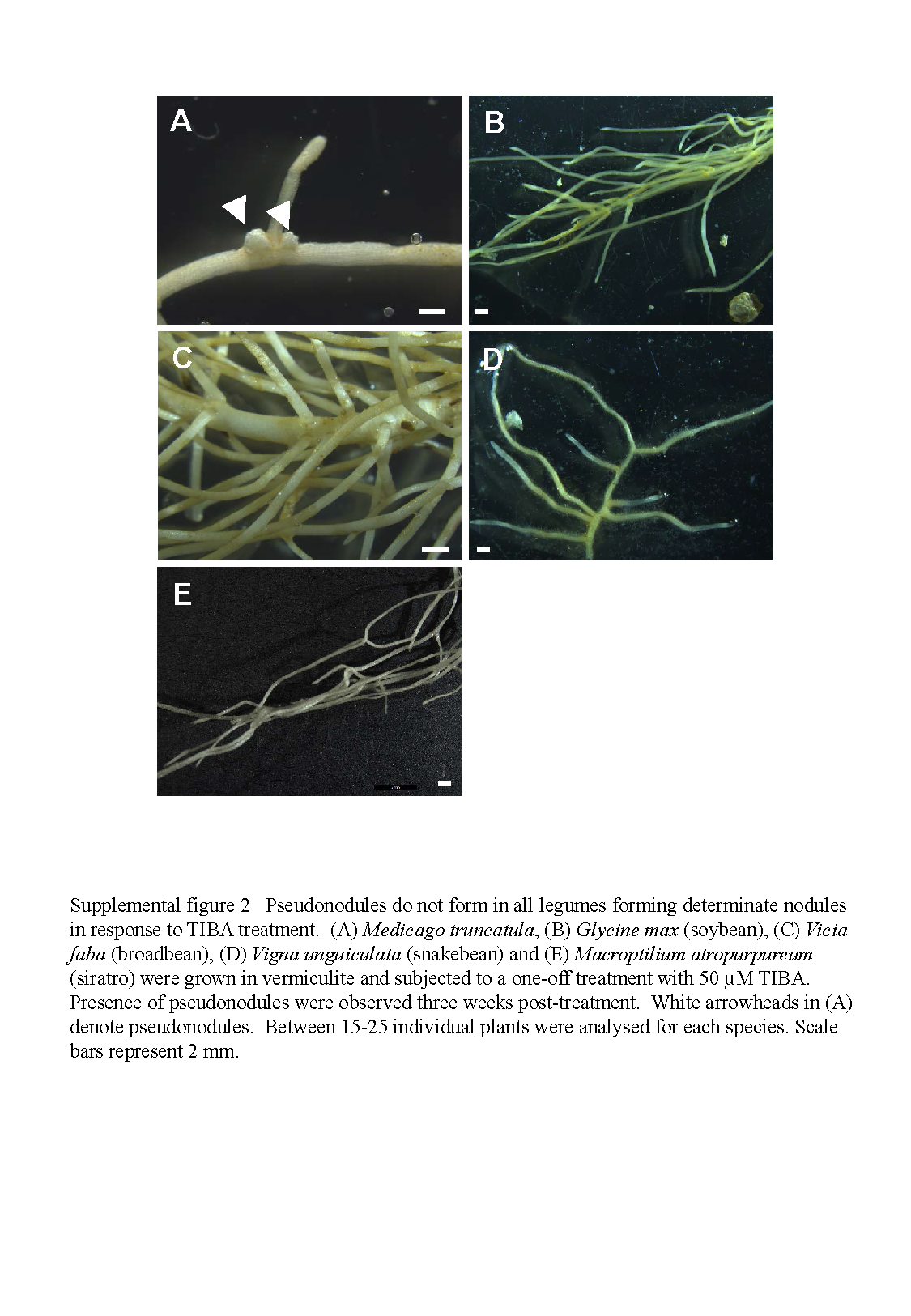

Supplement: Supplementary file 2 [file Image_2.TIF]

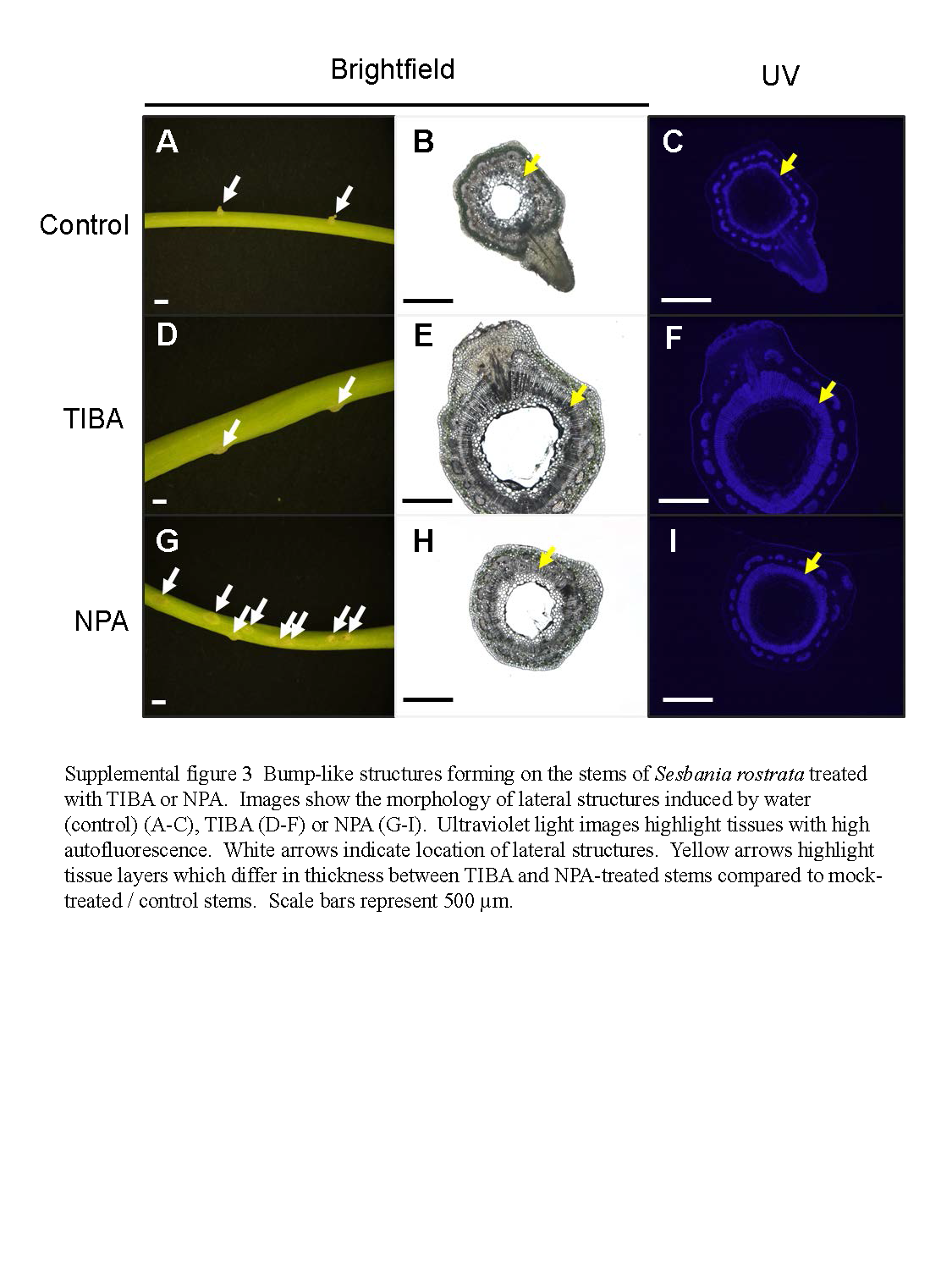

Supplement: Supplementary file 3 [file Image_3.TIF]

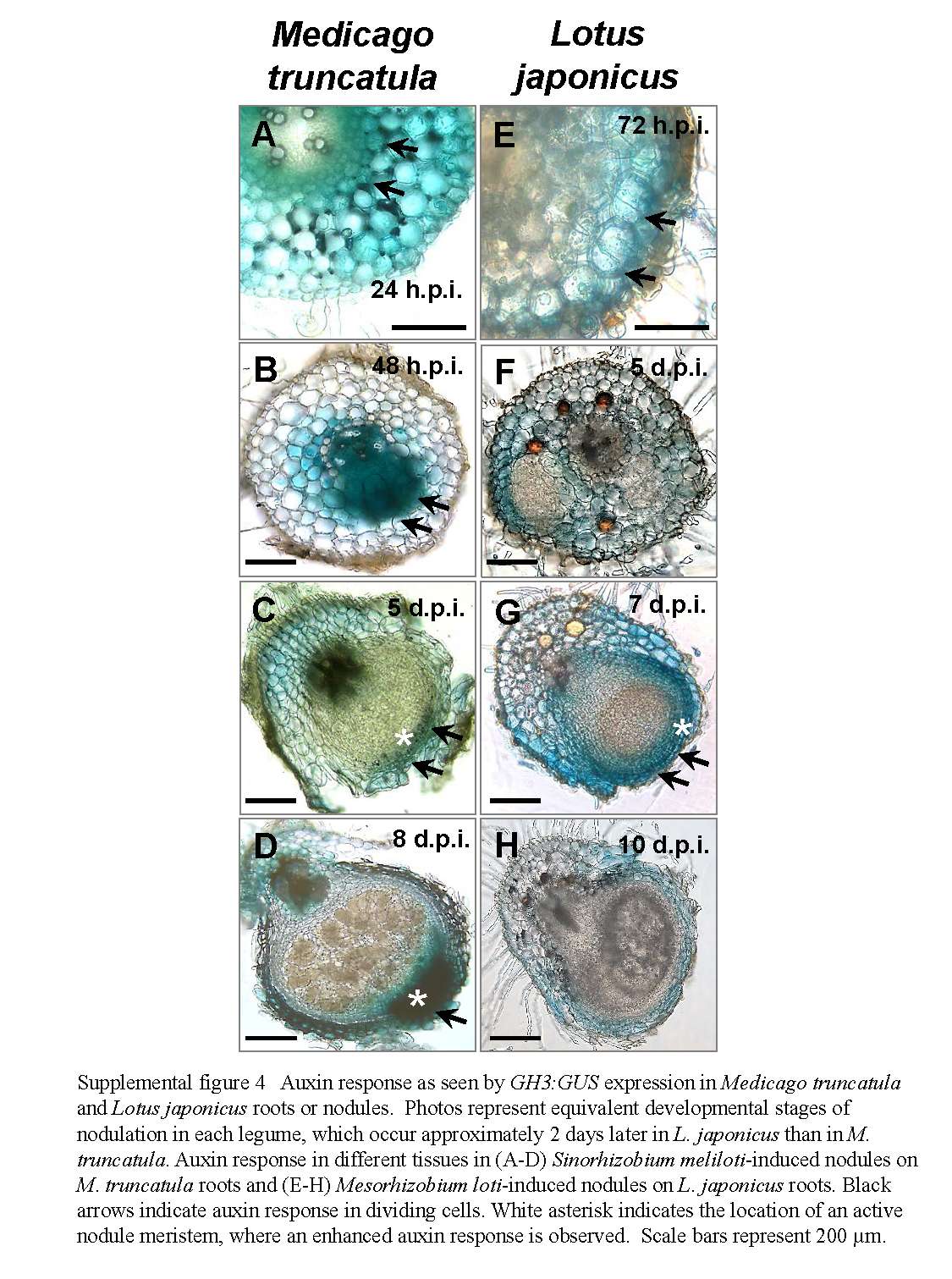

Supplement: Supplementary file 4 [file Image_4.TIF]

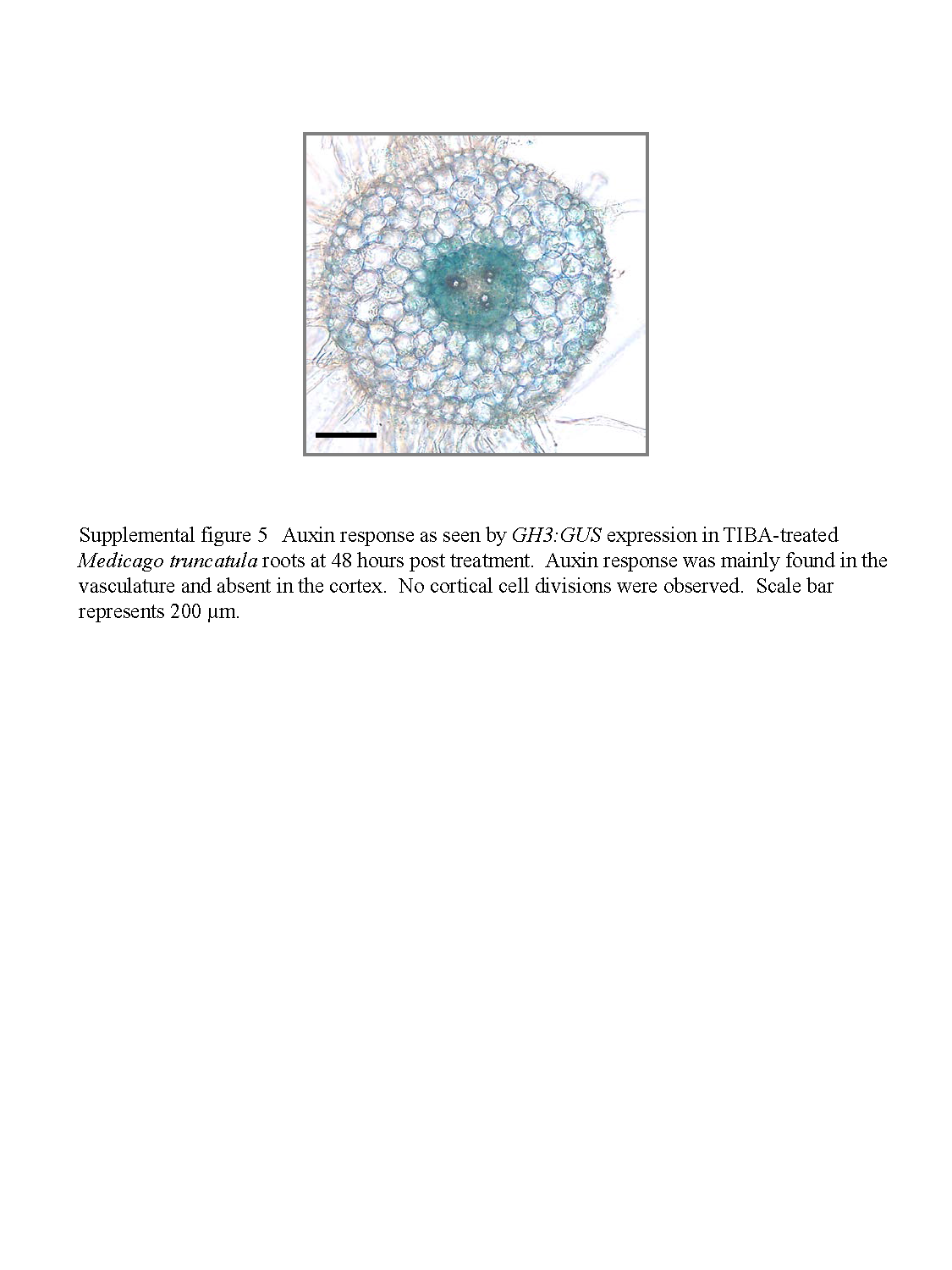

Supplement: Supplementary file 5 [file Image_5.TIF]

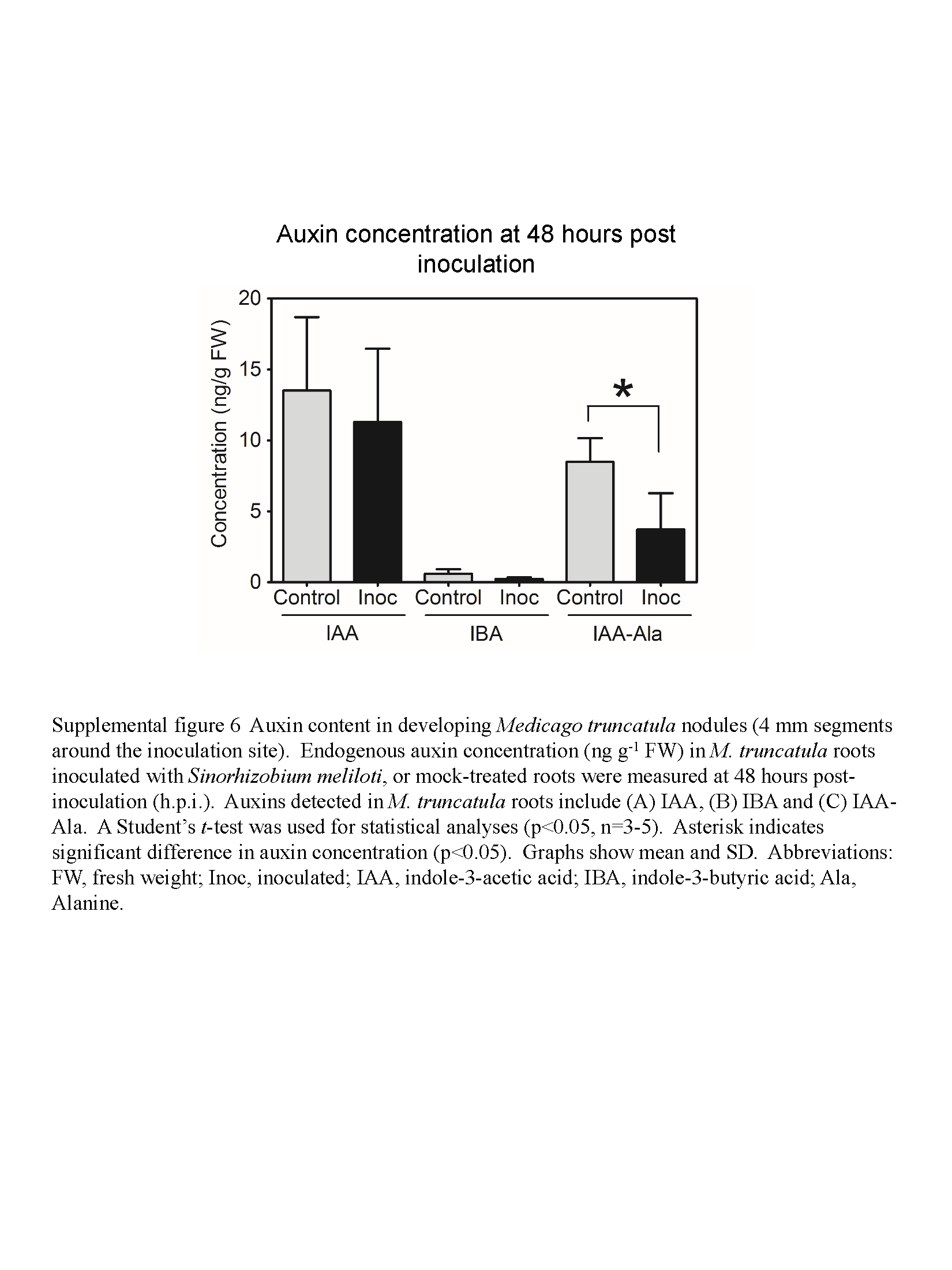

Supplement: Supplementary file 6 [file Image_6.TIF]
